# Genetics, epidemiology and management of clubfoot and related disorders

**DOI:** 10.1016/j.gendis.2025.101690

**Published:** 2025-05-17

**Authors:** Muhammad Umar, Liping Tong, Hongting Jin, Tamas Terebessy, Di Chen

**Affiliations:** a Research Center for Computer-aided Drug Discovery, Shenzhen Institutes of Advanced Technology, Chinese Academy of Sciences, Shenzhen, Guangdong 518055, China; b Faculty of Pharmaceutical Sciences, Shenzhen University of Advanced Technology, Shenzhen, Guangdong 518107, China; c The First College of Clinical Medicine, Zhejiang Chinese Medical University and Institute of Orthopaedics and Traumatology of Zhejiang Province, Hangzhou, Zhejiang 310053, China; d Centre for Translational Medicine, Department of Orthopaedics, Semmelweis University, Üllői út 26, Budapest 1085, Hungary; e Translational European-Asian Network (TEA-NET), Partner of Translational Medicine Working Group, Academia Europaea (AE), Üllői út 26, Budapest 1085, Hungary

**Keywords:** Associated genes pathways, Congenital anomaly, Congenitaltalipes equinovarus, Etiology of CTEV, Foot deformity

## Abstract

Clubfoot, medically termed congenital talipes equinovarus (CTEV), is a prevalent musculoskeletal birth defect, affecting approximately 0.3% of all live births. This serious congenital anomaly results from structural abnormalities in the foot and lower leg, leading to abnormal positioning of the ankle and foot joints. This review provides a comprehensive overview of the causative factors associated with CTEV and evaluates current therapeutic approaches. Although variations in genes encoding contractile proteins of skeletal myofibers have been proposed as contributors to the etiology of CTEV, no definitive candidate genes have been conclusively linked to increased risk. However, genes such as *TBX4*, *PITX1*, and members of the *HOXA, HOXC*, and *HOXD* clusters, as well as *NAT2*, have been implicated in the condition’s development, playing critical roles in limb development, muscle formation, and tissue differentiation. Also, Axin1 plays a key role in joint formation and skeletal development by inhibiting β-catenin-BMP signaling. It could significantly serve as a therapeutic target for fibular hemimelia and multiple synostoses syndrome. The exact mechanisms and the extent of their physical and genetic interactions remain subjects of ongoing research. Understanding the genetic determinants and cellular pathways involved in CTEV is crucial for unravelling the pathophysiology of this complex deformity.

## Introduction

Clubfoot, also known as talipes equinovarus (TEV) and connectively known as congenital talipes equinovarus (CTEV), is one of the most common congenital abnormalities in the musculoskeletal system with an incidence of 0.3 per 1000 live births and a gender ratio of 2.5 to 1 (male to female).^1^ This condition is characterized by the presence of forefoot and midfoot adductus, hindfoot varus, and ankle equinus, and appears as a rigid inward turning of the foot towards the midline of the body resulting in the obstinately contracted foot, which may affect one or both feet with bilateral cases accounting for 50 % of instances.^2^, ^3^, ^4^, ^5^ The presence of a clubfoot is typically evident at birth.^6^, ^7^, ^8^, ^9^

Analysis of CTEV serves to highlight the importance of understanding the etiologic principles associated with this common congenital disorder. In the minority of cases, CTEV is classified as secondary or syndromic, depending on clinical manifestations.^10^, ^11^, ^12^ Alternatively, in most cases (80 %), it may manifest isolated, resulting in the classification of idiopathic CTEV (ICTEV). The etiology of CTEV remains largely unknown, with secondary CTEV often manifesting in conjunction with distal arthrogryposis, congenital myotonic dystrophy, myelomeningocele, or other congenital disorders.^12^, ^13^, ^14^ Despite clinical similarities to the idiopathic form, secondary CTEV is thought to stem from neuromuscular and fetal abnormalities contributing to its pathogenesis, distinguishing it from ICTEV and syndromic CTEV in terms of clinical presentation, management, and proposed pathogenetic mechanisms.^15^

There are multiple factors such as maternal smoking, family history, twin or multiple fetuses, and women facing diabetes and obesity. The significant insights into the clubfoot’s pathogenesis are being revealed through genetic studies. Furthermore, the role of genetic factors in CTEV is also supported by a twin study where a higher risk was observed for identical twins rather than fraternal (33% *vs*. 3%). Depending upon the complex patterns of inheritance, CTEV is unlikely to be due to mutations in a single gene.^16^ There is evidence to suggest that the common disease-common variant hypothesis may apply to clubfoot. This hypothesis posits that the inheritance of common genetic variants, such as single-nucleotide polymorphisms (SNPs) with an allele frequency greater than 5%, each contributing a small increase in risk, collectively contributes to the susceptibility to clubfoot. Specifically, common genetic variants near *HOX* homeobox genes, insulin-like growth factor binding protein 3 (*IGFBP3*, MIM 146732), and caspase genes have been reported to be associated with isolated clubfoot. However, these variants are of relatively small effect and require replication in larger cohorts to confirm their significance in clubfoot susceptibility.

On the other hand, multiple synostoses syndrome (SYNS)^17^ is a disorder characterized by the abnormal fusion of multiple joints and fibular hemimelia (FH)^18^^,^^19^ is associated with lower limb disorder where the fibular bone is short or missing, causing various abnormalities such as bowing of the lower leg, clubfoot. Despite this, the precise signaling pathways involved in its development are not fully understood. Wnt/β-catenin signaling plays a crucial role in skeletal development, with Axin1 and Axin2 acting as key regulators within this pathway. To investigate their role in joint formation, researchers developed limb mesenchymal stem cell-specific *Axin1* conditional knockout (cKO) mice, as well as *Axin1* and *Axin2* double knockout (dKO) mice.^17^ Results declared that both *Axin1* cKO and *Axin1*/*Axin2* dKO mice exhibited joint developmental abnormalities, including joint tissue defects and fusion in multiple joints. These phenotypes closely resemble human SYNS, where synostoses (fusion) of the carpal and tarsal bones, as well as ankylosis (joint stiffening) of the elbow and knee, are observed, ultimately to cause CTEV. Importantly, the administration of β-catenin or bone morphogenetic protein (BMP) inhibitors significantly reduced joint fusion in *Axin1* cKO mice, suggesting a potential therapeutic pathway. This pathway is further elaborated in the text.^17^^,^^20^ These findings indicate that Axin1 plays an essential role in joint formation by regulating the β-catenin-BMP signaling axis. This makes Axin1 a potential therapeutic target for treating SYNS/FH-like phenotype by modulating these signaling pathways.^19^^,^^21^

This review investigates the intricate realm of CTEV by comprehensively exploring its underlying causes, genetic implications, and contemporary treatment modalities. Epidemiology sheds light on prevalence and hereditary and environmental factors, and opens avenues for further exploration into the genetic foundation of clubfoot. Various genes and mutations implicated in clubfoot development are scrutinized, along with emerging methodologies like genome-wide association studies, maternal influences, and teratogenic factors. Additionally, it elucidates the environmental contributions to clubfoot ailment. Detailed descriptions of current therapeutic approaches encompassing early detection, non-surgical interventions, and surgical methodologies are provided. This investigation provides a valuable resource for healthcare practitioners, researchers, and stakeholders who are committed to understanding and addressing this complex skeletal anomaly.

## Epidemiology of CTEV

### Prevalence and incidence of CTEV

Epidemiological studies are research investigations that examine the causes, patterns, and effects of health and disease conditions in specific populations. Numerous studies offer insights into the prevalence rates of clubfoot on a global scale.^22^, ^23^, ^24^ The estimated global prevalence of clubfoot stands at approximately 1–3 cases per 1000 live births based on information derived from multiple population-based studies and systematic reviews. Furthermore, in some previous studies, it was mentioned that the lowest prevalence was seen in China (3/1000 live births) and the highest prevalence was observed in Hawaiians and Moris (7/1000 live births).^25^ The prevalence rates of CTEV exhibit discrepancies across different global regions.^26^ Later, certain studies have highlighted higher prevalence rates in specific regions like Asia and Africa in comparison to North America and Europe.^22^^,^^27^^,^^28^ Genetic predisposition, environmental variables, and discrepancies in healthcare access can be linked to regional variances in the prevalence of CTEV. Asian people have higher prevalence rates, ranging from 1.11 to 4.39 per 1000 live births in nations like Malaysia, China, and India.^29^, ^30^, ^31^ Prevalence rates in African countries range from 1.11 to 4.54 per 1000 live births, with greater rates found in rural and underserved areas.^32^, ^33^, ^34^ These regions are especially vulnerable to healthcare resource constraints. On the other hand, prevalence rates in North American and European populations are typically lower, ranging from 0.5 to 2 per 1000 live births.^22^^,^^27^^,^^34^ Temporal patterns indicate variations in prevalence rates, which may be impacted by modifications to diagnostic standards, improvements in prenatal screening, and easier access to healthcare. There is a great deal of regional variation in Italy, where incidence rates vary from 2.8 to 23.4 per 1000 live births. Frequency rates range from 0.11 to 1.8 per 1000 live births, according to another research, some of which are from Europe, Australia, and Sweden. Some regions have shown a reduction in frequency over time.^24^^,^^35^^,^^36^

CTEV is a congenital deformity characterized by inward rotation of the foot, affecting mobility and quality of life if left untreated. However, the definition and diagnostic criteria for clubfoot can vary among physicians and healthcare centers, leading to differences in reported prevalence rates. Despite its relatively high prevalence, the genetic and molecular mechanisms underlying CTEV remain incompletely understood, presenting a challenge for both researchers and clinicians. Recent studies highlight significant variability in the reported prevalence of clubfoot due to differences in diagnostic criteria, prenatal detection, and study methodologies. A European population-based study (1995–2011) analyzing over 4.8 million births across 18 EUROCAT registries found a prevalence of 1.13 per 1000 births, with regional variations and an increasing prenatal detection rate from 4% to 16% over time.^37^ Similarly, an analysis of 549,931 births in Tuscany, Italy (1992–2011), reported a prevalence of 1.56 per 1000 births, with prenatal detection improving significantly over the two decades, suggesting that enhanced screening influences prevalence estimates.^38^ Additionally, a systematic review and meta-analysis of 48 studies from low- and middle-income countries found a wide prevalence range of 0.51–2.03 per 1000 live births, emphasizing that study design, diagnostic criteria, and reporting standards contribute to regional differences.^39^ These findings underscore the need for standardized diagnostic criteria and reporting methods to ensure accurate prevalence estimates and better public health planning.

## Genetics of CTEV

### Genes and proteins involved in CTEV

Genes and proteins are fundamental contributors to the development of skeletal tissues, cartilage, and muscle differentiation, playing a crucial role in CTEV deformity.^11^^,^^40^, ^41^, ^42^ BMPs, which are proteins involved in signaling pathways, regulate various processes such as cartilage, muscle, and bone differentiation, programmed cell death, as well as fracture repair of different genes mentioned in Table 1.

**Table 1:** Genes involved in CTEV/ICTEV.

Gene name	Gene expression	Chromosomal positions	Mutation or single-nucleotide polymorphism (SNP)	Reference
TNNT3	Role in skeletal muscle development or function, and provides instructions for making one form of a protein called troponin T	Chromosome 11	Mutation	122
TNNI2	Helps regulate muscle tension	Chromosome 11	Mutation	123
PITX1	Regulates the activity of genes to direct the shape and structure of tissues in limb development, including the bones, muscles, and tendons	Chromosome 5	SNP	42
ZC4H2	Linked to X-linked arthrogryposis; helps in the development of the nervous system during the early stages of human development	X-chromosome	Mutation	40
*MYH3*	Expressed as myosin-3, which is involved in muscle development and contractility; mutations in MYH3 and other related genes (like TPM2, TNNT3, TNNI2, and MYH8) have been identified to cause congenital contractures, including clubfoot, in distal arthrogryposis syndromes	Chromosome 17	Mutation	123
*MYH8*	Encodes a myosin protein involved in muscle development, and functions in skeletal muscle contraction	Chromosome 17	Mutation	123
*TNNC2*	Regulates muscle contraction and contributes to the pathogenesis of the condition	Chromosome 20	SNP	123
HOXD13	Potential role in limb development and patterning abnormalities associated with the condition	Chromosome 2	SNP	124
HOXC	Specify positional identity in the embryo rather than the development of any specific structure	Chromosome 12	Mutation	124
HOXD	Role in limb development and patterning	Chromosome 2	SNP	124
TBX3	Transcription factor implicated in limb development; induces biased differentiation of human-induced pluripotent stem cells into cardiac pacemaker-like cells	Chromosome 12	Mutation	125
TBX4	Involved in hindlimb development; role in aetiology of clubfoot; governs multiple processes during respiratory tract development, such as initial endodermal bud development, respiratory endoderm formation, and septation of the respiratory tract and oesophagus	Chromosome 17	Mutation	40
CASP9	Involved in apoptosis; role in regulating cell death pathways during limb development; essential for autophagosome maturation through regulation of mitochondrial homeostasis	Chromosome 1	Mutation	126
CASP10	Involved in apoptosis pathways; contributes to the pathogenesis of clubfoot	Chromosome 2	SNP	124
*COL9A1*	Encodes a collagen protein associated with cartilage development; involved in the aetiology of clubfoot	Chromosome 6	SNP	96
*SOX9*	Pivotal in skeletal development and cartilage formation; role in clubfoot pathogenesis	Chromosome 17	Mutation	127
*FLNB*	Encodes filamin B protein involved in cytoskeletal organization; involved in clubfoot pathogenesis	Chromosome 3	SNP	124
*CAND2*	Also known as TIP120B; involved in various cellular processes including transcriptional regulation	Chromosome 5	Mutation	128
*WNT7a*	Involved in embryonic development and limb patterning	Chromosome 3	Mutation	128
MTHFR	Involved in pathways related to embryonic development and folate metabolism	Chromosome 1	Mutation	96
HOXA9	Role in limb function, development, and patterning	Chromosome 7	SNP	129
HOXD12	Involved in limb development	Chromosome 2	SNP	124
HOXC13	Involved in limb development	Chromosome 12	SNP	124
CASP3	Involved in apoptosis	Chromosome 4	Mutation	126
CASP8	Involved in apoptosis; as a protease that promotes apoptosis	Chromosome 2	Mutation	124
COL1A2	Encodes collagen I protein; essential for bone and connective tissue formation	Chromosome 7	Mutation	40
TPM2	Encodes beta-tropomyosin; involved in muscle contraction	Chromosome 9	SNP	130
TPM1	Involved in muscle contraction	Chromosome 15	SNP	129
HOXD10	Implicated in limb development	Chromosome 2	SNP	124
HOXC11	Involved in limb development	Chromosome 12	SNP	42
HOXC12	Involved in limb development	Chromosome 12	SNP	124
*NAT1*	Encodes an enzyme involved in the metabolism of xenobiotics and carcinogens	Chromosome 8	SNP	126
*NAT2*	Encodes an enzyme involved in the metabolism of xenobiotics, its expression, and potential association with clubfoot	Chromosome 8	SNP	126
CFLAR	Encodes a protein involved in apoptosis regulation	Chromosome 2	Mutation	124
MYBPC1	Encodes a protein found in skeletal muscle	Chromosome 12	Mutation	126
APAF1	Encodes a cytoplasmic protein that initiates apoptosis	Chromosome 12	Mutation	126
BCL2	Encodes a protein involved in regulating apoptosis	Chromosome 18	Mutation	126
BID	Provides a connection between the death receptors; involved in apoptosis regulation	Chromosome 22	Mutation	126
NCOR2	Involved in transcriptional regulation	Chromosome 12	SNP	96
ZNF664	Encodes zinc finger protein involved in transcriptional regulation	Chromosome 7	SNP	96
FOXN3	Encodes a transcription factor	Chromosome 14	SNP	96
SORCS1	Encodes a protein involved in neuronal development and function	Chromosome 10	SNP	96
MMP7	Role in extracellular matrix remodelling	Chromosome 11	SNP	96

Despite numerous studies exploring the genetic pathways related to CTEV in recent years, a comprehensive understanding is still lacking, necessitating the identification of potential genes. Different genes have been identified that are associated with the syndromic clubfoot, as mentioned in Table 2. Various gene families have been discovered to indirectly influence this condition by overseeing limb formation, embryonic development, apoptosis, necrosis, morphogenesis, muscle contraction, and inflammatory processes.^40^

**Table 2:** Syndromic causes of clubfoot and associated genes in (idiopathic) congenital talipes equinovarus.

Syndrome name	Associated features	Genes identified	Reference
Van Maldergem syndrome 2	Facial dysmorphism, intellectual disability, growth retardation, eye abnormalities, skeletal abnormalities, hearing loss, heart defects, dental abnormalities, genital abnormalities	DCHS1, FAT4	131
TARP syndrome	Atrial septal defect, Robin sequence (Pierre Robin sequence), persistence of the left superior vena cava	RBM20	132
Schpritzen Goldberg syndrome	Weak muscle tone (hypotonia), heart or brain abnormalities, soft outpouching around the belly button	SKI	133
Saul-Wilson syndrome	Hearing loss, cataracts, blue sclerae	COG4	134
Santos syndrome	Intellectual deficit with hyperactivity, language delay, congenital hip luxation, short stature, kyphosis	WNT7A	135
Richieri-Costa-Pereira syndrome	Short stature, Robin sequence, cleft mandible, pre-/postaxial hand anomalies	EIF4A3	136
Recessive Larsen syndrome, Humero-spinal dysostosis, spondyloepiphyseal dysplasia	Prominent forehead, hypertelorism, depressed nasal bridge, flattened midface	CHST3	137,138
Recessive axonal Charcot-Marie-Tooth disease	Muscle weakness in legs, feet, arms, and hands, decreased muscle bulk, reduced tendon reflexes, sensory loss, foot and orthopedic problems	LMNA, GDAP1	139
Peroxisome biogenesis disorder 7A	Flattened face, broad nasal bridge, high forehead, widely spaced eyes (hypertelorism)	PEX26	140
Multiple synostosis syndrome	Proximal symphalangism of the fingers and/or toes is often associated with fusion of carpal and tarsal, humeroradial, and cervical spine joints	GDFS	88
Multiple epiphyseal dysplasia	Pain in the hips or knees after exercise, fatigue with long-distance walking	COL9A1, COL9A2, COL9A3, COMP, MATN3, SLC26A2	141,142
Mobius syndrome	Orofacial malformations, limb defects, musculoskeletal and cognitive abnormalities	PLXND1, REV3L	143,144
Marfan syndrome	Tall and thin with unusually long arms, legs, fingers, and toes	FBN1, TGFBR, TCFBR1, TGFBR2, SMAD3, TGFB2, SKI	145
Leoys-dietz syndrome	Craniosynostosis, scoliosis, pectus excavatum, pectus carinatum	TGFBR1, TGFBR2, SMAD3, TGFB2, TGFB3	133,145
Joubert syndrome	Broad forehead, arched eyebrows, eyelid ptosis, wide-spaced eyes, open mouth configuration, facial hypotonia	ATXN10, TCTN2	146
Epileptic encephalopathy	Electroencephalographic paroxysmal activity, seizures, cognitive, behavioral, and neurological deficits	AARS	147
Ehlers-Danlos syndrome, Vascular type	Arterial, intestinal, and uterine fragility, thin translucent skin, easy bruising	COL3A1	148
Ehlers-Danlos syndrome, Musculocontractural type 1	Distinctive craniofacial dysmorphism, congenital contractures of thumbs and fingers, clubfeet, severe kyphoscoliosis, muscular hypotonia, hyperextensible thin skin with easy bruising, wrinkled palms	CHST14	149,150
Ehlers-Danlos syndrome, Musculocontractural type 2	Joint dislocations and deformities, skin hyperextensibility, bruisability, fragility	DSE	151
Diastrophic dysplasia	Progressive abnormal curvature of the spine, hitchhiker’s thumbs	SLC26A2	152
Charcot-Marie-Tooth disease type 4D	Childhood-onset of severe, progressive, demyelinating sensorimotor neuropathy	NDRG1	153
Catel-Manzke syndrome	Bilateral hyperphalangy of the index finger, metacarpophalangeal joint, radial deviation of the index finger	TGDS	154
Carey-Fineman-Ziter syndrome	Bilateral facial weakness, Robin sequence, inability to fully abduct both eyes, facial dysmorphisms, muscle hypoplasia	MYMK	155
Bruck syndrome	Bone fragility and congenital joint contractures	PLOD2, FKBP10	156
Barth syndrome	Dilated cardiomyopathy, skeletal myopathy, neutropenia	TAZ	157
Autosomal dominant Larsen syndrome, recessive spondylocarpotarsal syndrome	Frontal bossing, midface hypoplasia, ocular hypertelorism	FLNB	158, 159, 160
Classic distal arthrogryposes	Inability to move small and large joints, hypoplastic muscles, soft tissue webbing over joints	TNNI2, TPM2, MYBPC1, MYH3	124,161
Freeman–Sheldon syndrome	Prominent forehead and brow ridges, midface hypoplasia, philtrum	MYH3	
Sheldon-Hall syndrome	Down slanting palpebral fissures, nasolabial folds	TNNI2, TPM2, MYBPC1, MYH3	
Distal arthrogryposis with ophthalmoplegia, ptosis, and retinal involvement	External ophthalmoplegia, pigmentary degeneration of retina, optic atrophy	PIEZO2, ECEL1	
Gordon syndrome	Hypertension, hyperkalemia, metabolic acidosis, normal renal function, low or normal plasma renin activity, elevated aldosterone concentration	PIEZO2	

Beyond the involvement of the β-catenin signaling pathway and Axin1 deletion in fibular hemimelia, other genetic factors contribute to this condition and related lower limb malformations. Variants in key developmental pathways, including WNT, BMP, and SHH signaling, have been implicated in skeletal abnormalities affecting limb formation. The WNT pathway is essential for limb patterning, while BMP signaling regulates chondrogenesis and bone growth. Additionally, disruptions in the SHH pathway, which controls anterior-posterior limb axis formation, have been linked to severe limb deficiencies, including hemimelia. These findings suggest that hemimelia arises from a complex interplay of genetic and molecular factors rather than a single gene mutation. Understanding these additional genetic influences provides deeper insight into the developmental mechanisms of lower limb malformations, offering potential avenues for future research and targeted therapeutic strategies.

### PITX1 and TBX4 pathway

**Figure 1:**
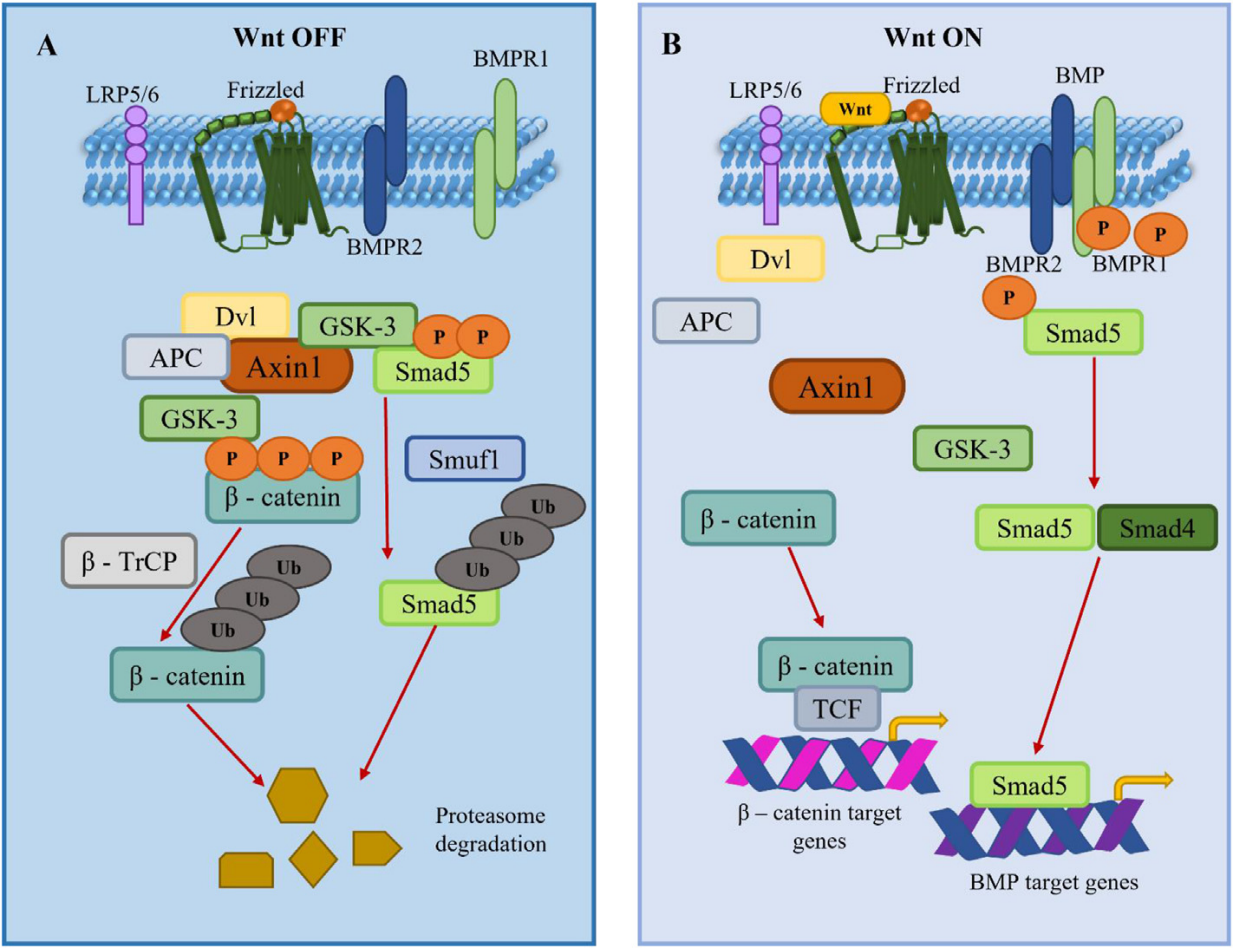
The mechanism of Pitx1 and TBX4 in CTEV. **(A)** Schematic representation for the divergence of an ancestral TBX4/5 transcription factor that has an activator domain (green) and for a gain of a repressor domain (purple) in TBX4. **(B)** TBX4 and TBX5 are hypothesized to influence limb bud growth, while the repressor domain specific to TBX is implicated in hindlimb specification. **(C)** The expression of TBX4 in hindlimbs is modulated by Pitx1, which may also play a role in regulating the expression of a potential co-repressor (CoR) necessary for the repressor function of TBX4. PITX1, paired-like homeodomain transcription factor 1; TBX4/5, T-box transcription factor 4/5; CTEV, congenital talipes equinovarus.

For early limb development, paired-like homeodomain transcription factor 1 (PITX1) and T-box transcription factor 4 (TBX4) transcriptional pathways are responsible. Many studies support the role of the PITX1-TBX4 developmental pathway in CTEV. Recent findings propose the involvement of PITX1 in clubfoot pathogenesis. Initially identified through a genome-wide linkage study in a five-generation clubfoot family with nine affected members, PITX1, a transcription factor from the bicoid-related homeodomain family, displayed significant linkage to the 5q31 locus with an LOD score of 3.31.^42^, ^43^, ^44^ A missense mutation (E310K) in PITX1 was identified within this family, segregating with clubfoot and absent in a control group of 500 individuals. This mutation, located in a conserved homeodomain region, leads to a dose-dependent reduction in wild-type PITX1 activity, indicating a dominant negative impact on transcription. Additionally, affected family members exhibited lower limb anomalies such as patellar hypoplasia, oblique talus, and preaxial polydactyly, suggesting a syndromic presentation linked to the mutation. Supporting this syndromic manifestation, PITX1 deletion was found in three individuals with isolated lower limb malformations, including polydactyly.^24^^,^^45^, ^46^, ^47^ The detailed possible pathways are shown in Figure 1.

TBX4, a direct target of PITX1, has been linked to the pathogenesis of clubfoot. Similar to PITX1, TBX4 exhibits specific expression in the hindlimb and plays a role in the formation of limb muscle and tendon patterns. Analysis of 66 familial clubfoot probands for genomic copy number variations unveiled a microduplication at 17q23.1q23.2 in three probands.^48^, ^49^, ^50^ This familial microduplication, identified with mild short-stature developmental hip dysplasia and subtle skeletal anomalies such as short, broad feet, demonstrated reduced penetrance. While it is considered a potential factor for familial isolated clubfoot, only a limited number of families have been associated with this microduplication. Another investigation detected a solitary multiplex family among 605 clubfoot families with a small 350 kb microduplication at 17q23.1q23.2, suggesting its correlation with clubfoot and short, broad feet, hence indicating variable expressivity. Therefore, further investigation is required to elucidate the involvement of TBX4 in the pathogenesis of isolated clubfoot, which seems to present as a syndromic phenotype.^51^^,^^52^ Moreover, it is essential to explore the potential interactive impact of genetic variations in the PITX1-TBX4 pathway shown in the most compelling genetic data points, towards the significance of the PITX1-TBX4 pathway in hindlimb development. Alterations in this pathway have been linked to isolated clubfoot phenotype, including a dominant segregating mutation in PITX1 and inherited TBX4 microduplications. Studies involving PITX1 mouse knockouts and copy number variations further strengthen this evidence.^40^^,^^53^

### Homeobox (HOX) gene

Patterns in limb and muscle are formed and influenced by the genes located in the HOXC cluster on chromosome 12q13.13, the HOXD cluster on chromosome 2q31.33, and the HOXA cluster on chromosome 7p15. In conjunction with the pathway involving PITX1-TBX4, HOX genes exhibit significant genetic associations with clubfoot phenotype. Studies revealed six significant chromosomal deletion regions (2q31-33, 3q23-24, 4p16-14, 7p22, 13q33-34, and 18q22-23), along with two duplication segments (6q21-27 and 10p15-11) that are specifically associated with clubfoot. Further investigation of the 2q31-33 deletion locus exposed correlations with variations in caspase 8 (CASP8), CASP10, and caspase 8 and Fas-associated death domain-like apoptosis regulator (CFLAR) genes, all of which play a role in the apoptotic pathway regulated by mitochondria.^24^^,^^40^^,^^47^ The discovery aligns with the pivotal role that apoptosis plays in the formation of limbs and muscles, as illustrated in Fig. 4A, which shows that posterior HOX genes are predominantly activated by canonical Wnt and canonical BMP pathways. In the presence of Wnt ligand, Wnt binds to the frizzled (FZD) receptor and low-density lipoprotein receptor-related protein 5/6 (LRP5/6) co-receptor and activates disheveled (DVL), leading to the inhibition of adenomatous polyposis coli (APC)/Axin/glycogen synthase kinase-3 beta (GSK-3β)-mediated β-catenin degradation. Phosphorylated SMAD1/5/8 through the canonical BMP pathway forms a complex with stabilized β-catenin and lymphoid enhancer binding factor 1 (LEF1) at the promoter region of posterior HOX genes and regulates their expression with the aid of Cdx family members. The finding that the more 3′ Hox genes function to specify the patterned identity of the more proximal limb structures, while the distal limb structures are specified by the more 5′ Hox genes. This is similar to the expression of these genes along the anterior-posterior axis of the animal. Mutations in this pathway can result in congenital abnormalities such as CTEV. Understanding the role of HOX genes in organ development and their involvement in clubfoot disease is important for the development of targeted gene therapy interventions.^54^

**Figure 2:**
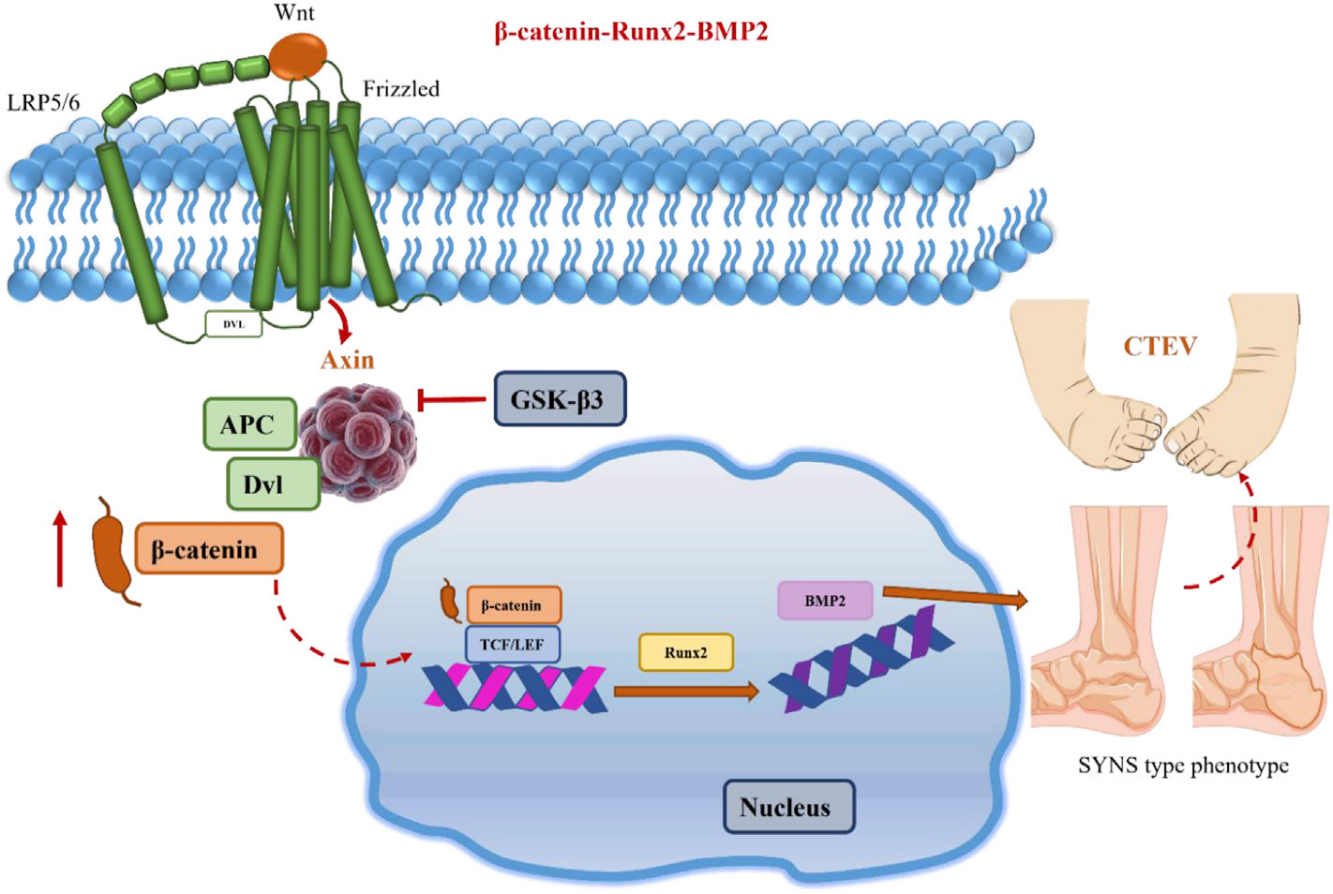
Morphogenic signaling pathways activating Hox genes along with molecular interactions and CTEV cellular dynamics in intrinsic apoptosis. **(A)** Phosphorylated SMAD1/5/8 through the canonical BMP pathway forms a complex with stabilized β-catenin and LEF1 at the promoter region of posterior HOX genes and regulates their expression with the aid of Cdx family members. Mutations in this pathway can result in congenital abnormalities such as congenital talipes equinovarus. **(B)** The induction of pro-apoptotic BCL2 family causes MOMP, leading to cell death. MOMP releases pro-apoptotic proteins such as CYCS and SMAC into the cytosol, where CYCS forms the apoptosome with APAF1, dATP, and pro-CASP9, activating CASP9. CASP9 then triggers executioner caspases CASP3 and CASP7, further supported by SMAC, which inhibits apoptosis blockers from the IAP family. BMP, bone morphogenetic protein; HOX, homeobox; CTEV, congenital talipes equinovarus; MOMP, mitochondrial outer membrane permeabilization; LEF1, lymphoid enhancer binding factor 1; BCL2, B cell lymphoma 2; CYCS, cytochrome c, somatic; SMAC, second mitochondria-derived activator of caspase; APAF1, apoptotic protease-activating factor 1; dATP, deoxyadenosine triphosphate; CASP3/7/9, caspase 3/7/9; pro-CASP9, pro-caspase 9; XIAP, X-linked inhibitor of apoptosis.

**Figure 3:**
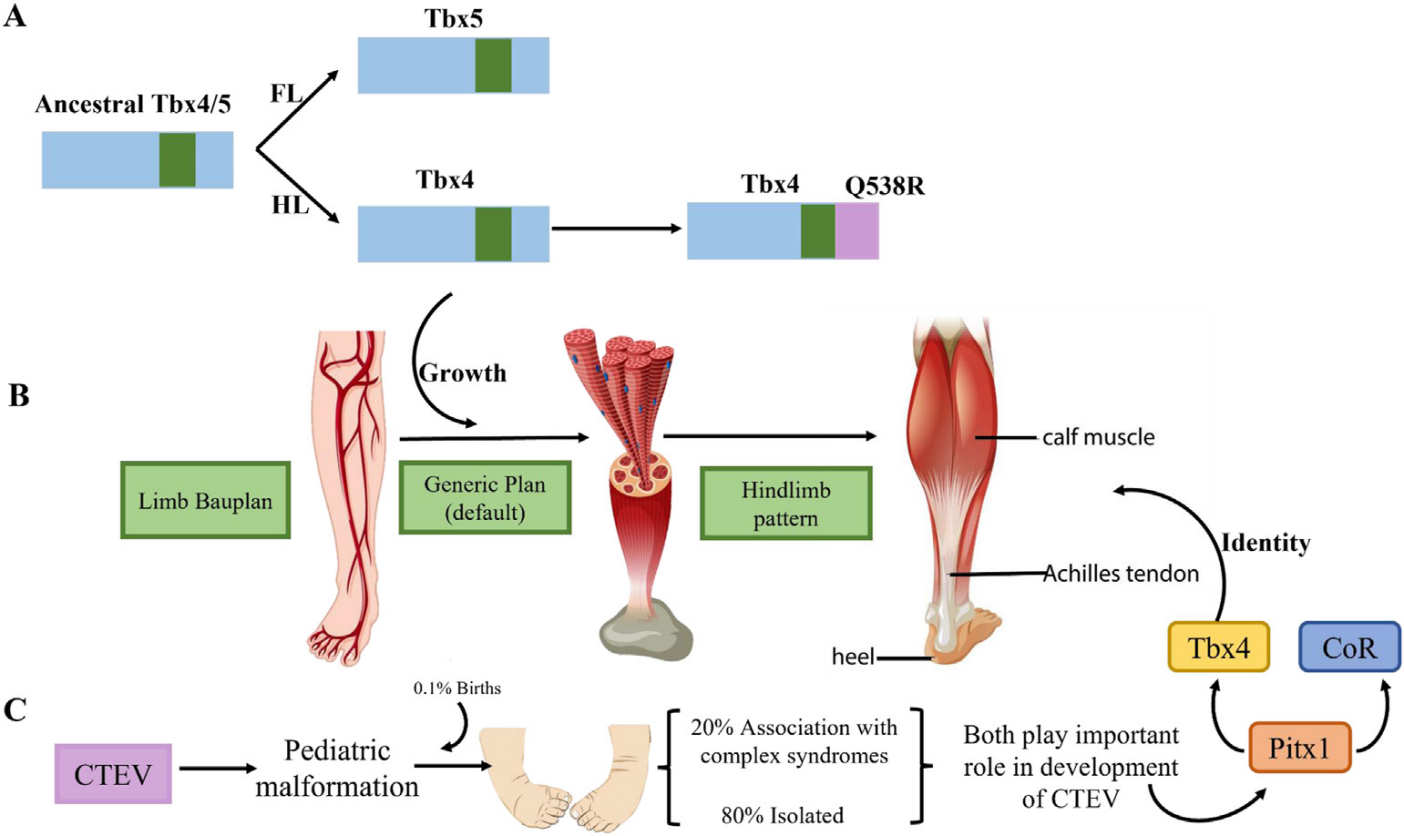
The schematic showing muscle structure at different levels of organization. **(A)** The thick and thin filaments are spaced in a symmetrical lattice. Every thick filament is surrounded by 6 thin filaments. **(B)** Muscle anatomy. **(C)** The thin filament includes actin, nebulin, tropomyosin, and the troponin complex (troponin I, T, and C). **(D)** The troponin complexes.

**Figure 4:**
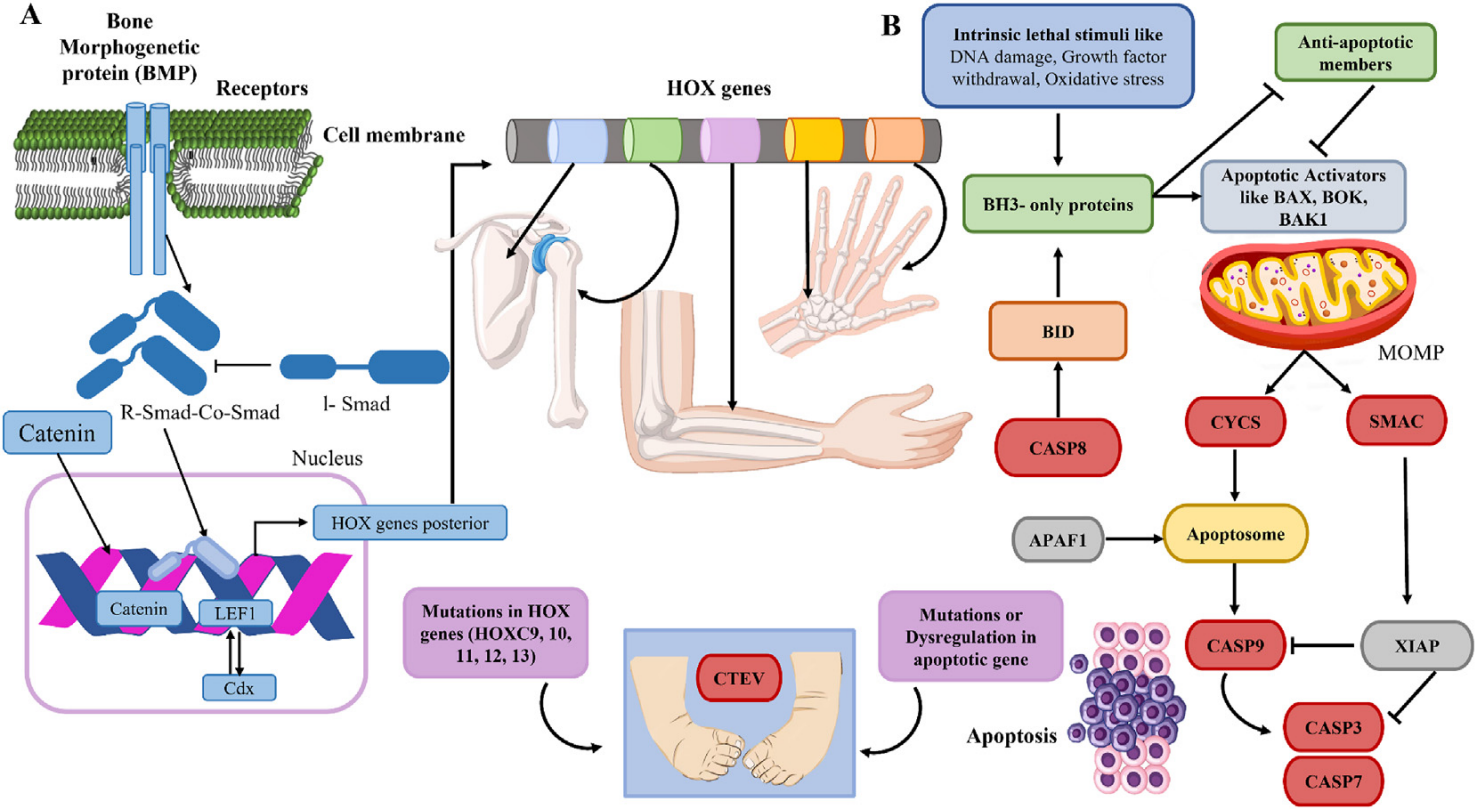
Model of integration of Wnt and BMP signaling pathways by Axin1. **(A)** In the absence of Wnt stimulation, β-catenin is degraded by the destruction complex. Smad5 is also degraded by the Axin1 destruction complex. **(B)** In the presence of Wnt ligands or the absence of Axin1, β-catenin and pSmad5 degradation are inhibited, resulting in the activation of both β-catenin and BMP/pSmad5 signaling. BMP, bone morphogenetic protein; APC, adenomatous polyposis coli; GSK-3, glycogen synthase kinase-3; DVL, disheveled; β-TrCP, beta-transducin repeats-containing protein; TCF, transcription factor.

Further investigation explored an additional five genes linked to apoptosis mediated by mitochondria, namely, CASP9, CASP3, apoptotic protease-activating factor 1 (APAF1), B cell lymphoma 2 (BCL2), and BH3-interacting domain death agonist (BID), revealing possible associations between genetic mutations in these genes and the occurrence of clubfoot.^55^, ^56^, ^57^ The deletion region of 2q31-33 encompasses the cluster of homeobox D genes (HOXD), which plays a crucial role in directing limb and muscle patterning during embryonic development. This cluster demonstrates functional overlap with the cluster of homeobox A genes (HOXA) located on chromosome 7p15.

Variations in SNPs within HOXD12-13 have been linked to idiopathic clubfoot.^58^, ^59^, ^60^ Furthermore, variations in the transmission rates of SNPs located in the HOXA cluster were observed in a case–control analysis, revealing interactions between HOXA and HOXD genetic variants as well as previously linked SNPs in genes related to mitochondrial-mediated apoptosis. Moreover, an investigation into an SNP in HOXA9 uncovered specific interactions with nuclear proteins and increased promoter activity, suggesting that alterations in the HOXA9 promoter region can impact gene expression, thus influencing the functional aspects of the isolated etiology of clubfoot. Recent findings have also highlighted HOXC microdeletions overlapping a noncoding region upstream of HOXC13, alongside a point mutation in HOXC11 segregating within a family affected by isolated clubfoot, and another point mutation in HOXC12 that is prevalent among clubfoot patients.^24^^,^^61^^,^^62^

### Apoptotic genes

Apoptotic genes such as CASP8, CASP10, and CFLAR, known for their role in the regulated cell death mechanism (Fig. 2A) and essential in the formation of the embryonic limb, had been previously correlated with microsatellite markers encompassing a deletion within the chromosomal locus 2q31-33 associated with congenital clubfoot. However, the subsequent analysis of 40 SNPs within seven apoptosis-related genes did not reveal any significant associations. Activation of intrinsic apoptosis can be initiated by a variety of internal or external signals, such as DNA damage, endoplasmic reticulum stress, oxidative stress, growth factor deprivation, or changes in microtubule dynamics. A crucial event in the intrinsic apoptosis pathway is the induction of pro-apoptotic factors belonging to the BCL2 family, namely BAX, BAK, and potentially BOK, leading to mitochondrial outer membrane permeabilization and eventual cell death. Mitochondrial outer membrane permeabilization triggers the release of pro-apoptotic proteins like CYCS (cytochrome c, somatic) and SMAC (second mitochondria-derived activator of caspase) from the mitochondrial intermembrane space into the cytosol. Subsequently, CYCS complexes with APAF1, deoxyadenosine triphosphate (dATP), and pro-CASP9 form the apoptosome, culminating in the activation of CASP9, which in turn activates the executioner caspases CASP3 and CASP7. The activation of executioner caspases is further facilitated by SMAC, which antagonizes or degrades inhibitors of apoptosis from the IAP family. Proper regulation of apoptosis is essential for normal limb development. Dysregulation of apoptosis-related genes can lead to genetic abnormalities such as CTEV. Understanding the role of apoptosis in clubfoot development provides insight into the underlying mechanisms and potential therapeutic targets for this condition.^7^^,^^45^^,^^47^^,^^63^

### Troponin to tropomyosin genes

The troponin (Tn) complex, crucial for regulating striated muscle contraction, consists of three subunits: Tn-I, Tn-T, and Tn-C. Tn-I inhibits actomyosin ATPase, Tn-T connects with tropomyosin and Tn-C, and Tn-C binds to calcium ions to counteract the inhibitory effects on actin filaments.^64^^,^^65^ A 2011 study involving NHW and Hispanic families identified specific SNPs in the troponin C2 (TNNC2) and tropomyosin 1 (TPM1) genes, suggesting their potential role in the development of ICTEV. TPM1, a member of the tropomyosin family, plays a role in both muscle contraction and the cytoskeleton of non-muscular cells. The presence of multiple SNPs in the TPM1 gene among ICTEV patients suggests that genes encoding contractile proteins may contribute to the condition.^66^^,^^67^

Distal arthrogryposis, a syndromic form of TEV, is characterized by mutations in genes that encode components of the muscle contractile complex, such as myosin heavy chain 3 (MYH3), TPM2, TNNT3, TNNI2, and MYH8, leading to muscle contractures. Although distal arthrogryposis and ICTEV share some clinical features, there are distinctions in their progression, indicating that different regulatory genes might be involved in ICTEV.^42^^,^^63^^,^^68^ A study by Gurnett and colleagues examined the TNNT3, MYH3, and TPM2 genes in ICTEV patients but found no significant association with clubfoot. Recent research has shown no major histological or cytological changes in muscle tissue after treatment (Ponseti method, casting, bracing, *etc*.). New approaches, including three-dimensional reconstruction and magnetic resonance imaging, are being explored to better understand muscle structure and the distribution of intramuscular fat, aiming to improve the assessment and treatment of ICTEV.^69^, ^70^, ^71^, ^72^ The pathway is shown in Figure 3.

### Methylenetetrahydrofolate reductase gene

In 2006, Sharp et al discovered that offspring carrying the 677T allele of the methylenetetrahydrofolate reductase gene (MTHFR) exhibit a reduced susceptibility to ICTEV. Subsequent research utilized complete genome sequencing to explore the allelic variations of MTHFR and the annexin A3 gene (ANXA3), revealing a distinct MTHFR allele unrelated to the one linked to clubfoot in the investigation conducted by Sharp et al.^47^^,^^71^^,^^73^ Examination through bioinformatic analysis indicated a potential modification in the protein-binding domain due to this genetic alteration (a shift in sequence: wild type being 264, mutant type being 267). Despite overlapping clinical presentations, these results suggest an association of the allele with a different hereditary condition rather than ICTEV. Moreover, distinct CNV patterns were detected in correlation with the affected specimens, underscoring the intricate nature of this familial disorder.^63^^,^^74^

### Dysplasia sulfate transporter gene

The gene known as the dysplasia sulfate transporter (DTDST) was proposed as a potential factor in the development of ICTEV and was examined. An investigation was conducted to determine if the R279W mutations were the underlying cause, however, no changes in the coding sequence were detected in 10 probands with ICTEV and a familial predisposition.^75^^,^^76^ It was concluded by the researchers that the prevalence of the R279W mutation was not significantly higher in the group of ICTEV probands compared with the control group.^77^^,^^78^

### Role of Axin1 in skeletal development

Axin1 plays a crucial role in skeletal development.^20^ A study conducted by Xie et al in 2022 defines the essential role of Axin1 in lower limb development.^18^ CTEV/ICTEV are congenital conditions also related to lower limb development, resulting in clubfoot.^19^ Therefore, this study was found to be highly associated in this context. Researchers demonstrate through their findings that specific deletion of *Axin1* in mesenchymal cells of the limb can result in a tarsal condition, FH-like phenotype. It was evident in the *in vivo* study that Axin1 not only controls limb development through the canonical β-catenin pathway but also BMP signaling. The study was conducted on mice as a subject, and it was elaborated that inhibition of BMP signaling and β-catenin pathway could significantly converse with the FH phenotype.^18^^,^^79^

FH is associated with complete or partial deletion of the fibular bone, resulting in moderate shortening of the femur, bowing lower legs, curved tibia, deficiency of important soft tissues, ankle and knee joint instability, *etc*. The equinovalgus position of the foot is witnessed.^80^ The canonical Wnt signaling pathway, which is conserved throughout evolution, plays a crucial role in many biological processes, especially during development and in maintaining tissue health. A key aspect of this pathway is how it controls β-catenin, a protein that acts as a downstream effector. The regulation of β-catenin happens through a destruction complex in the cytoplasm, with Axin1 being a central scaffold protein in this complex. Axin1 directly interacts with all the other core components and is considered the rate-limiting factor in regulating β-catenin signaling.^81^ However, studying the role of Axin1 in skeletal development and tissue maintenance has been challenging because mice with Axin1 mutations die early in embryonic development (around day E9.5). Despite this, research in both humans and mice has shown that Wnt/β-catenin signaling is vital for all major aspects of skeletal development, including the formation of the skull, limbs, and joints.^82^

Another important signaling pathway involved in skeletal development is the BMP pathway. Both Wnt and BMP pathways have overlapping functions in controlling skeletal development and maintaining bone hemostasis^18^ as shown in Figure 4. However, the key question remains: How do these two pathways work together to regulate skeletal development and ensure bones and joints stay in normal function over time?

Axin1 is crucial for integrating Wnt and BMP signaling pathways, which are key to limb development and bone health. Without Wnt signaling, both β-catenin and Smad5 proteins are degraded through a complex involving Axin1. However, when Wnt ligands are present or Axin1 is absent, this degradation stops, activating β-catenin and Smad5 signaling. While Wnt signaling stabilizes β-catenin, it is unclear how it also stabilizes pSmad5 through Axin1. BMP signaling also acts downstream of β-catenin during lower limb development. Inhibiting β-catenin rescues the hindlimb and forelimb phenotype in *Axin1*-deficient mice, indicating that β-catenin is upstream of BMP signaling during limb development. FH disease may result from genetic and environmental factors, such as exposure to harmful conditions during early pregnancy, which could temporarily boost β-catenin-BMP signaling in limb cells. However, the exact environmental triggers and genetic mechanisms are still unknown. In summary, Axin1 is a key regulator of lower limb development, and disruptions in Axin1 or related signaling pathways during early development may contribute to FH. This research has significant implications for diagnosing and treating FH.^17^^,^^18^^,^^21^^,^^83^

### Axin-β-catenin-BMP2 signaling pathway

Msultiple SYNS is a condition marked by the progressive fusion of multiple joints (*i.e.*, CTEV), often leading to conductive hearing loss in affected individuals.^84^ Mutations in key genes involved in the BMP and fibroblast growth factor (FGF) signaling pathways, such as noggin (NOG), growth differentiation factor 5 (GDF5), GDF6, and FGF9, have been identified as the underlying cause of the disease. These genetic alterations disrupt normal BMP and FGF signaling, critical for joint development. Despite the known significance of BMP signaling in skeletal and joint formation, the potential role of Axin/β-catenin signaling in SYNS development has yet to be explored. The specific involvement of Axin/β-catenin in the onset and progression of SYNS remains uncertain.^85^, ^86^, ^87^, ^88^, ^89^ It was also reported that deletion of *Axin1* results in many other bone deformities, such as cartilage defects,^20^ the osteoarthritis-type phenotype in the temporomandibular joint,^21^ and defects in postnatal bone growth.^90^

**Figure 5:**
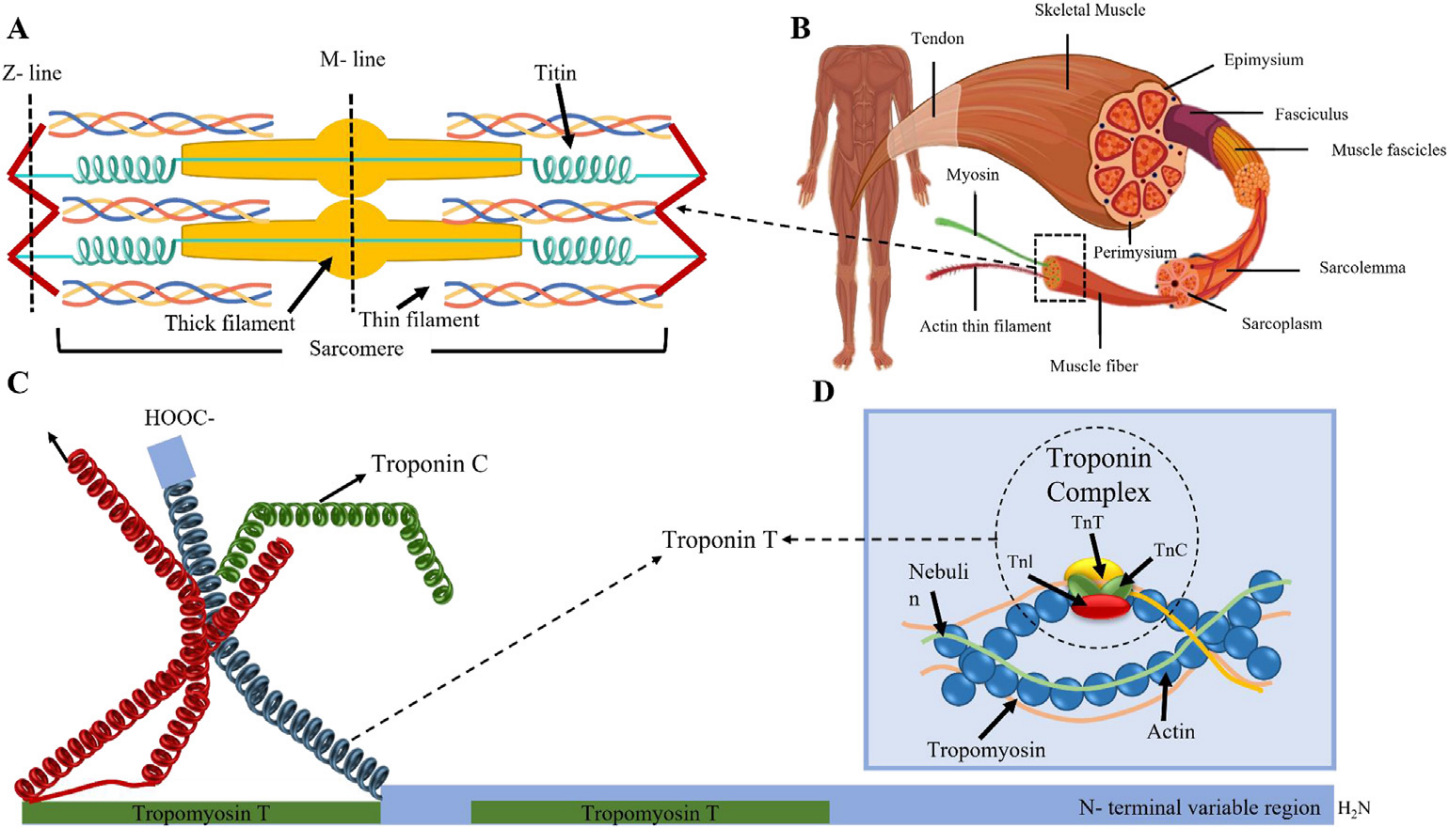
β-catenin-BMP2 signaling activation caused by inhibition of Axin1 could lead to SYNS-like phenotype. BMP2, bone morphogenetic protein 2; GSK-3β, glycogen synthase kinase-3 beta; APC, adenomatous polyposis coli; DVL, disheveled; TCF, transcription factor; LEF, lymphoid enhancer binding factor; Runx2, RUNX family transcription factor 2; SYNS, multiple synostoses syndrome; CTEV, congenital talipes equinovarus; LRP5/6, low-density lipoprotein receptor-related protein 5/6.

It was reported in previous studies^17^ that Axin1 collaborated with GSK-3β to induce β-catenin degradation and thus negatively regulated its functioning, and the deletion of *Axin1* led to an increase in the expression of β-catenin protein. We have previously performed a study to elucidate the role of β-catenin in upper limb development. For this purpose, pregnant *Axin1*^*Prrx1*^ cKO mice at the E9.5 stage were treated with a single dose of 2.5 mg/kg iCRT14 (β-catenin inhibitor, intraperitoneal injection). It is known that iCRT14 disrupts the interaction between β-catenin and transcription factor 4 (TCF4) and specifically inhibits β-catenin-induced gene transcription. These results further support the notion that β-catenin activation is a major signaling event when the Axin1 gene is deleted, which means it significantly regulates the formation of joints through the β-catenin pathway.^17^ It has been observed that BMP signaling plays an important role in synovial formation of joints. On the other hand, the BMP signaling pathway is probably one of those immediate downstream pathways of Axin1/β-catenin signaling during skeletal development. Transfection of Axin1 siRNA up-regulated RUNX family transcription factor 2 (Runx2) and BMP2 expression. Co-transfection of Axin1 and Runx2 siRNA could inhibit BMP2 expression induced by Axin1 siRNA, suggesting that Axin1 siRNA-induced BMP2 expression was mediated by Runx2. Figure 5 shows that β-catenin-BMP2 signaling activation caused by inhibition of Axin1 could lead to SYNS-like phenotype in mice.^83^^,^^91^^,^^92^

Significantly, this skeletal defect phenotype can be partially rescued by the inhibition of β-catenin or BMP signaling. These findings imply that the temporal and transient activation of β-catenin-BMP signaling is the key event in SYNS pathogenesis. This novel revelation holds promise for the development of new therapeutic strategies aimed at effectively treating this genetic disease.^17^

## Other techniques and approaches to gene identification in CTEV

### Candidate gene association study

Candidate gene association studies have been instrumental in revealing common genetic variants linked to clubfoot susceptibility, such as HOX homeodomain genes and caspase genes, which demonstrate moderate associations.^42^ It is noteworthy that HOXD12 and HOXD13 have emerged as pivotal susceptible genes for idiopathic CTEV. More specifically, SNPs situated in the 5′-flanking sequence of the HOXD12 gene (rs847154) and exon 1 of the HOXD13 gene (rs13392701) are correlated with idiopathic CTEV. Furthermore, an SNP within the HOXA9 gene in conjunction with CASP10 has implications in apoptosis among simplex clubfoot cases.^42^

Further genotyping of 40 SNPs in seven apoptosis-related genes, including CASP10, in 210 simplex trios and 139 multiplex families has confirmed that variations within these genes may play a role in the development of clubfoot. An association has also been established between CTEV and MTHFR polymorphisms. Transmission disequilibrium was observed in 84 nuclear pedigrees for SNPs (rs592121, rs1135056) within the collagen type IX alpha 1 chain (COL9A1) gene, with elevated expression of COL9A1 detected in clubfoot patients compared with unaffected individuals (*t* = 4.7500; *p* < 0.05), indicating its potential significance as a susceptibility gene for clubfoot development.^58^^,^^73^^,^^93^

### Genome-wide association studies

Genome-wide association studies (GWAS) have identified a significant link between isolated clubfoot and a specific SNP (rs7969148) situated between the nuclear receptor corepressor 2 (NCOR2) and zinc finger protein 664 (ZNF664) genes on chromosome 12q24.31 ^42^. Furthermore, this study has pinpointed SNPs near the genes forkhead box N3 (FOXN3), sortilin-related VPS10 domain containing receptor 1 (SORCS1), and matrix metalloproteinase 7 (MMP7)/transmembrane Protein 123 (TMEM123) in cases of clubfoot. These discoveries have been replicated in subsequent studies involving a collective of 766 cases and 1363 controls.^93^ The determination of sample size efficacy and the necessary statistical power in genetic association studies is dependent on various factors such as the number of markers, the assumed inheritance mode (recessive/dominant model), and the study configuration (case/control or case/parent trio).^44^ Nevertheless, it is essential to highlight that this GWAS did not unveil any significant genetic associations with CTEV from common variants that sustained significance post-correction for genome-wide significance. Hence, larger sample sizes will be imperative for forthcoming investigations. Given the often-perceived complexity of clubfoot inheritance, with more than 75% of cases lacking a family history, conducting a comprehensive GWAS could potentially unveil significant insights.^61^^,^^94^

### Next-generation sequencing techniques

Next-generation sequencing, which includes whole-genome sequencing and whole-exome sequencing, has revolutionized the landscape of gene discovery associated with diseases and is increasingly utilized in clinical diagnosis.^95^ Despite the widespread adoption of next-generation sequencing, whole-exome sequencing has emerged as particularly prominent due to its cost-effectiveness and improved bioinformatics capabilities, proving to be a valuable diagnostic instrument for investigating undiagnosed genetic conditions. Especially in scenarios where traditional linkage analysis proves inadequate, whole-exome sequencing has exhibited effectiveness in identifying disease-related genes.^25^^,^^46^^,^^95^, ^96^, ^97^

In a recent investigation, Yang et al performed whole-exome sequencing on a family with six individuals affected by clubfoot. Their analysis revealed a heterozygous mutation in the filamin B (FLNB) gene (c.4717G > T; p.D1573Y) as the probable pathogenic mutation responsible for the condition. Additionally, by screening 53 more clubfoot patients, they detected two extra missense mutations (c.1897A > G; p.M633V) and (c.2195A > G; p.Y732C) within the FLNB gene.^98^

## Contemporary management and treatments of CTEV

### Early diagnosis and evaluation

Assessment methods differ between pediatric and adult populations. In young children, evaluation entails clinical observation, radiographic measurements, and functional assessments. Clinical observation involves scrutinizing standing, gait, and at times, running patterns. Ankle movements are particularly assessed using a goniometer to determine foot position and range of motion. Evaluating subtalar motion can be complex and is often evaluated subjectively. Forefoot adduction and supination are commonly evaluated by obtaining a weight-bearing image on a glass plate.^8^^,^^99^^,^^100^

Radiographic measurements in children aged 3–4 years necessitate standardization involving weight-bearing foot and tibia positioning, with the X-ray beam inclined at 30°. Both anterior-posterior and lateral views are acquired, encompassing traditional metrics such as the Beatson Combined Talo-Calcaneal angle (TC index) and the Talo-first metatarsal angle (T-MT-1 angle). Nevertheless, drawing definitive conclusions regarding anatomical relationships from radiographs in young children presents challenges due to the prevalence of cartilaginous structures over bony ones. Several grading systems, including those employing a 100-point scale, have been established by researchers. These systems evaluate foot dimensions, morphology, range of motion, radiographic criteria, and functional parameters like pain and mobility. Laaveg and Ponseti introduced functional evaluation criteria emphasizing satisfaction, pain, weight-bearing function, passive motion, and gait analysis.^7^

## Treatments

### Non-surgical interventions

The Ponseti method is one of the most famous methods of treating clubfoot without surgery. The Ponseti method, involving manipulation, casting, Achilles tendon tenotomy, and foot abduction bracing, has emerged as the predominant treatment globally for idiopathic clubfoot.^99^ Ignacio Ponseti initiated and perfected his methodology for treating clubfoot in the latter part of the 1940s, driven by the realization that extensive surgical procedures often resulted in enduring foot pain and deformities. The development of the Ponseti technique, emerging from a quest for a more efficient and less intrusive option, originated from extensive research on the functional and pathological anatomy of both typical and clubfoot-afflicted feet.^101^ The Ponseti protocol involves a specific series of manipulations, cast applications, and Achilles tendon tenotomy to achieve the correction of clubfoot. Effective communication with families right from the start concerning the casting and bracing regimen is essential, given that the treatment necessitates a commitment spanning a minimum of 4 years. Ideally, treatment should commence within the initial weeks of life, entailing gentle foot manipulations in a clinical setting, followed by successive application of long leg casts as per Ponseti’s guidelines.^102^ Although plaster casting is preferred for its flexibility, fiberglass materials have also demonstrated efficacy in achieving correction. Cast changes are executed every 5–7 days. Within the Ponseti methodology, the rectification of the cavus deformity precedes the management of hindfoot varus, forefoot adduction, and hindfoot equinus through successive casting sessions. A tenotomy of the Achilles tendon is frequently required to address any residual equinus contracture, typically carried out under local anesthesia for younger patients and formal sedation for older children.^103^ After the tenotomy, the foot is placed in abduction with minimal dorsiflexion in the final cast. Continuous parental education and assistance, encompassing problem-solving and range of motion exercises, are vital for long-term success. Despite these measures, relapses of clubfoot persist as a challenge, frequently linked to intolerance towards the brace. Older children presenting with specific deformities may necessitate customized interventions such as tibialis anterior tendon transfers.^104^, ^105^, ^106^, ^107^, ^108^

An alternative to extensive surgical procedures for clubfoot treatment is the French method, also known as the functional method, which involves daily manipulation of the infant’s clubfoot by a skilled physiotherapist, followed by immobilization through adhesive taping to maintain the correction achieved via stretching.^109^ Unlike the Ponseti method, which utilizes casting, the French method employs taping to keep the foot in the corrected alignment while permitting a degree of mobility.^110^ Furthermore, the French technique emphasizes the significance of enhancing the peroneal muscles to maintain long-term correction. In the 1990s, a continuous passive motion device was integrated into the treatment regimen to facilitate additional stretching while asleep. Initially, treatments are administered daily for the first two months, followed by a reduction in frequency to three times per week until the child reaches six months of age.^111^^,^^112^ After achieving successful correction, caregivers are instructed to continue with home exercises and employ night splints until the child begins walking. Variability is observed in the reported success rates of the French approach. Dimeglio et al reported a success rate of 74% in averting surgical intervention using this technique. However, other studies have indicated a higher incidence of surgical intervention following the implementation of the French method, particularly for posterior release procedures aimed at addressing residual equinus. A notable drawback of the French method is the significant time commitment demanded from parents as children undergo daily formal physical therapy for two months.^113^^,^^114^

### Surgical interventions

The progression of surgical treatments for clubfoot initiation commenced with advancements in aseptic methodology and anesthesia. Lister introduced antiseptic principles in 1867, while Esmarch detailed a flat-rubber bandage for blood extraction from limbs in 1873. The utilization of the pneumatic tourniquet by Cushing in 1904 further ameliorated limb surgery. Radiography enabled accurate evaluation of deformities, and the advent of anesthesia signaled the conclusion of the surgical rebirth, enabling orthopedic surgery to evolve into a scientific field.^115^^,^^116^

Tendon transfers gained prominence in the 1920s, with Dunn introducing the tibialis anterior tendon transfer to prevent relapse. Garceau and Manning observed positive results with the tibialis anterior transfer in instances of recurring deformity. Barr emphasized the necessity of maintaining muscle equilibrium when contemplating tendon transfers. Simultaneously, surgical procedures focusing on foot skeletal structures were developed. Lund, Agustoni, and Morestin conducted talectomies in efforts to rectify foot positioning, while Jones advocated for osteotomies and wedge resections. Denis Browne suggested crescentic tarsal resection, although Jones cautioned against extensive bone removal due to potential functional limitations.^117^^,^^118^

Contemporary trends indicate that clubfoot poses a surgical dilemma, with mild cases potentially correctable through manipulation and immobilization. Nonetheless, prolonged manipulation and casting attempts often yield unsatisfactory outcomes for severe cases. While surgical interventions stress the importance of early alignment for reinstating normal skeletal and soft tissue structure, a consensus on the timing, extent, and assessment criteria for surgery remains elusive. The absence of long-term follow-up studies on surgically treated cases contributes to this uncertainty. Consequently, inadequate correction of the initial deformity, coupled with the emergence of severe iatrogenic deformities, persists. Immediate restoration of the anatomic position of displaced bones is impeded by the necessity of wire fixation through joint cartilage, inevitably resulting in cartilage and capsule damage, and subsequent joint rigidity. Additionally, surgical interventions often lead to significant scarring, a particular concern in infants. Moreover, the average failure rate of clubfoot surgery stands at 25 %, accompanied by a range of complications including wound issues, persistent forefoot supination, loss of reduction and recurrence, overcorrection of the hindfoot, dorsal subluxation of the navicular, compromised mobility of the ankle and subtalar joints.^119^, ^120^, ^121^

## Conclusion

CTEV or clubfoot, is a multifaceted congenital deformity with significant implications for affected individuals if left untreated. Despite its prevalence and the advancements in clinical management, the underlying molecular mechanisms and genetic contributors remain incompletely understood, presenting a challenge to both researchers and clinicians. This review has explored the genetic basis of CTEV, highlighting both common and rare genetic variants that contribute to the etiology and disease conditions. The debate between the common disease-common variant hypothesis and the common disease-rare variant hypothesis underscores the complexity of the genetic landscape of CTEV. While common variants near genes such as HOX, IGFBP3, and CASP have been associated with CTEV, their modest effect sizes necessitate further validation in larger cohorts. Conversely, rare variants in the PITX1-TBX4 pathway, which plays a crucial role in hindlimb development, provide deeper insights into the genetic mechanisms of CTEV. Notably, TBX4 dosage sensitivity suggests that genetic predisposition to CTEV involves both common polygenic risk and rare, highly penetrant mutations. Moreover, the β-catenin signaling pathway plays a fundamental role in limb development and bone structure, contributing to congenital limb abnormalities. In this context, the role of Axin1 was discussed in detail, as its deletion has been specifically linked to CTEV, FH, and SYNS. This evidence reinforces the connection between β-catenin dysregulation and these phenotypic manifestations. By integrating these genetic and molecular insights, this review provides a comprehensive foundation for future research, offering a unified platform to explore targeted therapeutic approaches.

Furthermore, the analysis of non-surgical treatments, particularly the Ponseti method, reaffirms the efficacy of conservative management in the majority of cases, yet it also highlights the need for personalized treatment plans, especially in cases resistant to standard interventions. The review also underscores the importance of understanding the biomechanical and histological changes in muscle tissue associated with CTEV, suggesting that muscle contractile protein abnormalities may contribute to the persistence or recurrence of the deformity, even after initial successful treatment. Critically, the review points out the gaps in our current understanding of the pathophysiology of CTEV. While significant strides have been made in identifying potential genetic and molecular targets, the precise mechanisms by which these factors interact to produce the clinical phenotype remain elusive. The lack of consistent histological abnormalities in muscle tissue post-treatment (Ponseti method, bracing, casting, surgical treatment) suggests that CTEV may involve more subtle, possibly epigenetic, modifications that are not yet fully understood. In the end, the review emphasizes that future therapeutic approaches should not only focus on correcting the deformity but also consider the underlying genetic and molecular disruptions.

## CRediT authorship contribution statement

**Muhammad Umar:** Writing – original draft, Conceptualization. **Liping Tong:** Writing – review & editing. **Hongting Jin:** Writing – review & editing. **Tamas Terebessy:** Writing – review & editing. **Di Chen:** Validation, Conceptualization.

## Funding

This project was supported by the 10.13039/501100012166National Key Research and Development Program of China (No. 2022YFA1207500), the 10.13039/501100001809National Natural Science Foundation of China (No. 82394445, 82161160342, 82030067 to D.C.), and the Shenzhen Science and Technology Innovation Bureau (Guangdong, China) (No. LCYSSQ20220823091402005-E-001) to D.C.

## Conflict of interests

The authors of this paper declare that no competing interests are involved for all of them in this study.
